# Secondary structures involving the poly(A) tail and other

**DOI:** 10.15698/mic2014.04.140

**Published:** 2014-04-05

**Authors:** Zarmik Moqtaderi, Joseph V. Geisberg, Kevin Struhl

**Affiliations:** 1 Department of Biological Chemistry and Molecular Pharmacology, Harvard Medical School, Boston, MA 02115, USA.

**Keywords:** mRNA isoforms, mRNA stability, polyU element, poly(A) tail, mRNA structure, Saccharomyces cerevisiae

## Abstract

In *Saccharomyces cerevisiae*, previous measurements of mRNA stabilities have been determined on a per-gene basis. We and others have recently shown that yeast genes give rise to a highly heterogeneous population of mRNAs due to extensive alternative 3’ end formation. Typical genes can have fifty or more distinct mRNA isoforms with 3’ endpoints differing by as little as one and as many as hundreds of nucleotides. In our recent paper [Geisberg *et al.* Cell (2014) 156: 812-824] we measured half-lives of individual mRNA isoforms in *Saccharomyces cerevisiae* by using the anchor away method for the rapid removal of Rpb1, the largest subunit of RNA Polymerase II, from the nucleus, followed by direct RNA sequencing of the cellular mRNA population over time. Combining these two methods allowed us to determine half-lives for more than 20,000 individual mRNA isoforms originating from nearly 5000 yeast genes. We discovered that different 3’ mRNA isoforms arising from the same gene can have widely different stabilities, and that such half-life variability across mRNA isoforms from a single gene is highly prevalent in yeast cells. Determining half-lives for many different mRNA isoforms from the same genes allowed us to identify hundreds of RNA sequence elements involved in the stabilization and destabilization of individual isoforms. In many cases, the poly(A) tail is likely to participate in the formation of stability-enhancing secondary structures at mRNA 3’ ends. Our results point to an important role for mRNA structure at 3’ termini in governing transcript stability, likely by reducing the interaction of the mRNA with the degradation apparatus.

We used the anchor-way method to deplete RNA Polymerase II from the nucleus, causing rapid cessation of transcription without the adverse physiological consequences typically encountered in traditional temperature-shift polymerase shutoff methods. Using direct sequencing (involving no sample amplification or library construction) of the polyadenylated RNA remaining in the cells at various time points after the polymerase depletion, we obtained stability measurements for the individual 3’ mRNA isoforms from each gene. Cumulatively, these individual half-lives were used to determine an average half-life for the mRNAs arising from each gene.

Two interesting patterns emerged from this gene-averaged analysis. First, gene length is inversely correlated with mRNA stability. Second, transcripts of genes in certain functional classes have notably short (ribosome biogenesis; RNA helicases) or unusually long (oxidative phosphorylation) half-lives. This could be biologically relevant under shifting physiological conditions, providing a mechanism for co-regulation of gene groups needed in response to particular situations.

When considered individually, the mRNA isoforms arising from a single gene can exhibit surprising heterogeneity in their stabilities. It is not unusual for mRNA isoforms from the same gene to vary substantially in stability, often by more than two-fold. In most instances, the different mRNA isoforms arising from adjacent or closely-spaced polyadenylation sites tend to have similar half-lives, but in 259 cases, mRNA isoforms from polyadenylation sites only one nucleotide apart have half-lives that differ by more than two-fold. In such isoform pairs, there is a strong statistical preference for the more stable isoform to end in a U. Similarly, in isoforms arising from closely spaced (<=5 nt) poly(A) sites, there is a marked preference for the more stable isoform to be U-rich in these last 5 residues.

Groups of mRNA isoforms terminating at nearby positions frequently have similar half-lives. We refer to such groupings of isoforms as "clusters" arbitrarily defined as terminating over a region spanning no more than 30 nt, with no more than a 10 nt gap between consecutive isoforms, and in which no member has a stability differing by more than two-fold from that of any other member. A cluster’s half-life is defined as the average half-life of all isoforms contained within it, and it may or may not be similar to those of other clusters in the same 3’ untranslated region (UTR). When half-lives of consecutive clusters are compared, the intervening sequence between them has one of three properties: it may be stabilizing (if the downstream cluster is more stable than the upstream cluster), destabilizing (if the downstream cluster is less stable than the upstream cluster), or neutral (if the two clusters have similar half-lives) (Figure 1A). By these definitions, we found 560 stabilizing elements and 851 destabilizing elements in the yeast genome.

**Figure 1:**
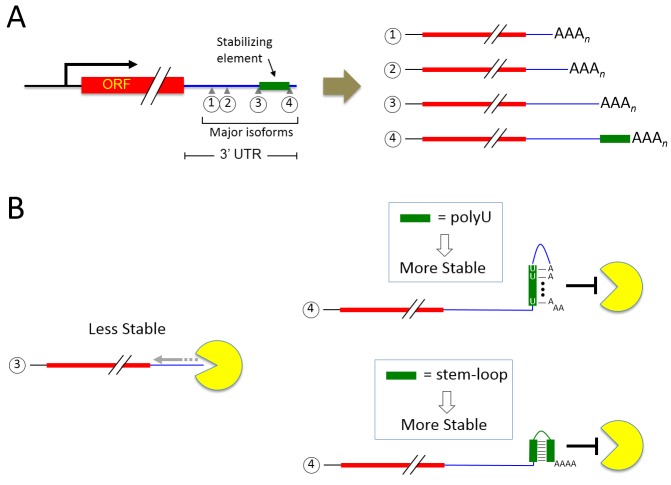
FIGURE 1: **(A)** A schematic depicting a typical yeast gene whose transcription profile is made up of multiple clusters (numbered from 5’ to 3’). Major mRNA isoforms within clusters are indicated by small gray triangles. A stability element (green rectangle) is present in major isoform 4 but absent in isoform 3 and shorter mRNA species. **(B)** Major isoform 4 possesses a longer half-life than isoform 3 due to the presence of a stability element. In this model, isoform 3 has no stability element and is readily degraded by the exosome complex (yellow Pac-Man shape) from the 3’ to 5’ direction. The presence of either a polyU-poly(A) tail structure or a stem-loop near the 3’ terminus of isoform 4 blocks exosome-dependent degradation.

A search for common sequence motifs in stability-influencing elements yielded only one specific sequence motif—a 20 nt polyU stretch found in ten percent of stabilizing elements. It is possible that other stability-influencing sequence elements might function by forming secondary (or higher order) structures with sub-regions of the RNA sequence that lie outside of 3’ UTRs and might thus be specific to individual genes. Alternatively, other elements might contain binding sites for stability-influencing RNA-binding proteins, many of which function only when the underlying sequence adopts a specific three-dimensional fold. In either case, such motifs encoded within stabilizing and destabilizing elements would be exceedingly difficult to identify by conventional motif discovery algorithms, as they don’t take into account potential RNA folding and long-range interactions.

The nature of the computationally-identified polyU sequence motif immediately suggested a model by which the polyU element might be involved in forming a hairpin structure with the poly(A) tail. Structural modeling of mRNA isoforms terminating before and after the polyU motif clearly predicts the formation of stem-loop structures between the polyU elements and poly(A) tails in the more stable mRNAs terminating downstream of (and therefore containing) the polyU element. In five out of six instances, incorporation of the polyU element into stability-neutral inter-cluster sequences had the remarkable ability to confer stabilization on the mRNA cluster terminating downstream.

From the above, it is clear that the poly(A) tail itself may participate in secondary structures that confer increased stabilization. In light of this, we modeled the predicted structures of isoforms arising from adjacent poly(A) sites but possessing different stabilities. These nearly-identical isoforms have different degrees of predicted double-strandedness in their poly(A) tails. The degree to which the poly(A) tail is predicted to participate in double-stranded structures is directly correlated with the relative stabilities of the isoforms.

The suggestion from the above experiments is that 3’ UTR secondary structure elements involving poly(A) tails can directly influence mRNA isoform stability. We also examined the more general influence of termini-proximal 3’ UTR structures by introducing G-C-rich sequences predicted to form stable stem-loops not involving the poly(A) tail. When substituted for normally neutral inter-cluster sequences, these G-C-rich stem-loop forming sequences, much like the polyU motif, also had a significant stabilizing effect on isoforms of the downstream cluster.

We propose that structural features in 3’ UTRs of mRNAs are important determinants of transcript stability. These structural features may exist in several different forms. First, poly(A) tail-containing structures appear to form stem-loops with polyU elements. Second, non-poly(A)-containing stem-loops and other structures, as described here and as also seen near the 3’ termini of yeast histone mRNA transcripts, appear to enhance stability. Third, higher order structures such as pseudoknots or triple helices have also been shown to dramatically stabilize a number of mammalian and viral transcripts.

How might strong secondary structures near the termini of mRNAs enhance stability? In yeast, most mRNAs are degraded by one of major pathways: by the Xrn1 or Rat1 exonuclease in the 5’ to 3’ direction, or by the multi-subunit exosome complex in the 3’ to 5’ direction. In either case, it is thought that deadenylation of poly(A) tails by the major cytoplasmic deadenylase Ccr4-Pop2-Not complex or by the Pan2-Pan3 duo is the first step in targeting the mRNA into the degradation pathway. The yeast poly(A) binding protein Pab1 is believed to inhibit transcript turnover by associating with poly(A) sequences longer than ~ 10-12 bases; transcripts with shorter poly(A) tails are rapidly degraded.

We used RNA immunoprecipitation to determine Pab1 occupancy levels at mRNA isoforms either containing or lacking stabilizing sequences. In mRNA isoforms containing natural or transplanted stabilizing sequences, Pab1 occupancy is markedly decreased compared to the levels at isoforms lacking stabilizing sequences, suggesting that structural features of the mRNA may inhibit the ability of the poly(A) tail to bind Pab1.

The implication from the experiment above is that mRNA secondary structures at 3’ termini inhibit the binding of Pab1 due to their double-stranded nature. We reasoned that perhaps the double-stranded nature of the termini of these isoforms also blocks the association of the mRNA degradation machinery. We examined a recently published set of genome-wide RNA binding protein data from the Tollervey laboratory, initially focusing on Pab1 occupancy at naturally-occurring polyU-containing loci. In accord with our immunoprecipitation studies, we found that Pab1 occupancy at these loci was drastically reduced at regions immediately downstream of the polyU elements. Similarly, Ski2 (an exosome-associated helicase important for 3’ to 5’ mRNA degradation) was also nearly absent in mRNA isoforms containing polyU elements. The absence of Ski2 at the polyU-containing isoforms led us to propose a model whereby structures at mRNA 3’ termini provide a protective barrier against degradation by effectively preventing the association of components of the degradation machinery (Figure 1B), resulting in increased isoform stability.

The biological importance and prevalence of the differential stabilities of 3’ UTR isoforms have only recently begun to be uncovered. In zebrafish embryos, maternally contributed mRNA isoforms are generally more stable than *de novo* transcribed variants, likely due to the presence of stability-enhancing motifs at 3’ termini. Conversely, shorter, more stable mRNA isoforms result in the activation of multiple proto-oncogenes in cancer cells as compared to their non-transformed counterparts, likely due to loss of miRNA-mediated repression. Taken together with our genome-wide mapping of hundreds of stabilizing and destabilizing elements, these observations make it increasingly evident that regulation of isoform-specific stability is an important mechanism by which cells can fine-tune gene expression programs to suit environmental conditions.

